# Vitamin D Status of HIV-Infected Women and Its Association with HIV Disease Progression, Anemia, and Mortality

**DOI:** 10.1371/journal.pone.0008770

**Published:** 2010-01-19

**Authors:** Saurabh Mehta, Edward Giovannucci, Ferdinand M. Mugusi, Donna Spiegelman, Said Aboud, Ellen Hertzmark, Gernard I. Msamanga, David Hunter, Wafaie W. Fawzi

**Affiliations:** 1 Department of Epidemiology, Harvard School of Public Health, Boston, Massachusetts, United States of America; 2 Department of Nutrition, Harvard School of Public Health, Boston, Massachusetts, United States of America; 3 Department of Medicine, Brigham and Women's Hospital and Harvard Medical School, Boston, Massachusetts, United States of America; 4 Department of Internal Medicine, Muhimbili University of Health and Allied Sciences, Dar es Salaam, Tanzania; 5 Department of Biostatistics, Harvard School of Public Health, Boston, Massachusetts, United States of America; 6 Department of Microbiology and Immunology, Muhimbili University of Health and Allied Sciences, Dar es Salaam, Tanzania; 7 Department of Community Health, Muhimbili University of Health and Allied Sciences, Dar es Salaam, Tanzania; 8 Department of Global Health and Population, Harvard School of Public Health, Boston, Massachusetts, United States of America; Lerner Research Institute, United States of America

## Abstract

**Background:**

Vitamin D has a potential role in slowing HIV disease progression and preventing mortality based on its extensive involvement in the immune system; however, this relationship has not been examined in large studies or in resource-limited settings.

**Methodology/Principal Findings:**

Vitamin D levels were assessed in 884 HIV-infected pregnant women at enrollment in a trial of multivitamin supplementation (not including vitamin D) in Tanzania. Women were followed up for a median of 69.5 months, and information on hemoglobin levels, HIV disease progression, and mortality was recorded. Proportional hazard models and generalized estimating equations were used to assess the relationship of these outcomes with vitamin D status.

**Conclusions/Significance:**

Low vitamin D status (serum 25-hydroxyvitamin D<32ng/mL) was significantly associated with progression to WHO HIV disease stage III or greater in multivariate models (incidence rate ratio [RR]: 1.25; 95% confidence intervals [CI]: 1.05, 1.50). No significant relationship was observed between vitamin D status and T-cell counts during follow-up. Women with low vitamin D status had 46% higher risk of developing severe anemia during follow-up, compared to women with adequate vitamin D levels (RR: 1.46; 95% CI: 1.09, 1.96). Women in the highest vitamin D quintile had a 42% lower risk of all-cause mortality, compared to the lowest quintile (RR: 0.58; 95% CI: 0.40, 0.84). Vitamin D status had a protective association with HIV disease progression, all-cause mortality, and development of anemia during follow-up in HIV-infected women. If confirmed in randomized trials, vitamin D supplementation could represent a simple and inexpensive method to prolonging the time to initiation of antiretroviral therapy in HIV-infected patients, particularly in resource-limited settings.

## Introduction

Human Immunodeficiency Virus (HIV) disease is characterized by a progressive deterioration in immune function. Interventions that offset this impairment have the potential to slow HIV disease progression and improve quality of life. The latter has become increasingly important in the face of HIV infection becoming a chronic disease in those with access to anti-retroviral therapy (ART).

Vitamin D may represent one such intervention with its extensive involvement in the human immune response. Vitamin D has several important metabolic functions, including regulation of calcium movement in gastrointestinal mucosa, renal tubules, and the skeleton [1]. An increase in the occurrence of infections in children with rickets, the classic vitamin D deficiency disease has been reported, although the mechanisms implicated are not fully understood [2]. Laboratory studies have suggested an important role of vitamin D in immune regulation; the discovery of vitamin D receptors (VDRs) on peripheral blood mononuclear cells has fuelled the interest in vitamin D as an immune modulator [3]. More recently, vitamin D has been shown to have an integral role in innate immunity and response to infections such as tuberculosis [4], [5], [6].

Few studies have examined the relationship of vitamin D with HIV disease progression or survival [7]; one small longitudinal study in Norway found that HIV-infected patients with low 1,25-dihydroxyvitamin D_3_ [1,25(OH)_2_D] levels, the biologically active metabolite of vitamin D, had significantly shorter survival time than those with normal concentrations [8]. The Trial of Vitamins [9] conducted in Tanzania, in which HIV-infected pregnant women were supplemented with multivitamins (not including vitamin D) and followed up to observe pregnancy outcomes and disease progression, provides an opportunity to expand our knowledge on vitamin D status and health, with particular relevance to HIV infection.

## Methods

### Ethics Statement

Verbal informed consent was obtained from all participants due to the low rates of literacy in Tanzania and the procedure is detailed in an earlier publication [10]. The study protocol was approved by the Research and Publications Committee of the Muhimbili University College of Health Sciences, the Ethical Committee of the National AIDS Control Program of the Tanzanian Ministry of Health, and the Institutional Review Board of the Harvard School of Public Health.

### Study Population

The study design has been previously described in detail [10]. Briefly, participants were HIV-infected pregnant women enrolled in a randomized, double-blind, placebo-controlled trial of vitamin supplementation (1995–1997). Pregnant women (12–27 weeks gestation) were randomized to receive one of four regimens from enrollment until at least 18 months after delivery: i) vitamin A alone (30mg β-carotene, 5000 IU preformed vitamin A); ii) multivitamins including vitamin A (30mg β-carotene, 5000 IU preformed vitamin A, 20mg B_1_, 20mg B_2_, 100mg B_3_, 25mg B_6_, 50µg B_12_, 500mg C, 30mg E, 0.8mg folic acid); iii) multivitamins excluding vitamin A; or placebo. At delivery, women in vitamin A groups were given an additional oral dose of vitamin A (200,000 IU), whereas women in the other two groups received a placebo. All women received iron-folate daily and chloroquine weekly as malaria prophylaxis, according to national guidelines for antenatal care at the time the trial was conducted. At the time of the study, ART was not available to most women in Tanzania, including participants in the trial.

### Assessment of Baseline Covariates

Structured interviews were conducted during the baseline visit to collect information on demographic characteristics including age, educational level, and money spent on food per day. Data were also collected on morbidities, symptoms, and hospitalizations during the current pregnancy. Study physicians conducted a complete medical examination and collected blood, urine, stool, and vaginal swab specimens. HIV disease stage was classified in accordance with the World Health Organization (WHO) guidelines [11]. Anthropometric measurements were obtained by trained research assistants using standardized procedures and calibrated instruments.

### Assessment of Outcomes

Women were followed through monthly study clinic visits, where physicians performed a clinical examination, and a nurse assessed self-reported symptoms and HIV-related complications in the preceding period. HIV disease stage was assessed at each visit, in accordance with WHO criteria [11]. Women who missed a clinic visit or travelled out of Dar es Salaam were followed *via* a home visit and neighbors or relatives were asked about vital status. Verbal-autopsy techniques were used to ascertain the cause of death, by conducting standardized interviews with relatives and reviewing medical records.

### Laboratory Methods

Blood samples were obtained from study participants at enrollment (12–27 weeks of gestation), and plasma was stored at or below −70°C. Maternal vitamin D status was assessed using serum levels of 25-hydroxyvitamin D [25(OH)D]. Serum 25(OH)D levels were measured by the fully automated chemiluminescence ADVANTAGE 25(OH)D assay system obtained from Nicholas Institute Diagnostics, San Juan Capistrano, CA. Absolute CD4, CD8, and CD3 T-cell counts were evaluated with the FACSCount system (Becton-Dickinson, San Jose, CA). Hemoglobin levels were assessed using either a CBC5 Coulter Counter (Coulter Corporation, Miami) or by the cyanmethemoglobin method with use of a colorimeter (Corning Inc, Corning, NY). HIV-1 serostatus was determined by the Enzygnost anti-HIV-1/2 Plus assay (Dade Behring, Germany) followed by the Wellcozyme HIV-1 recombinant test (Murex Biotech Ltd, Dartford, UK). Discordant results were resolved using Western blot test (Bio-Rad Laboratories Ltd., Hertfordshire, UK) [12].

Sera and genital swabs were collected to test for candidiasis and sexually transmitted infections (STIs) including syphilis, gonorrhea, and trichomoniasis. Malaria parasites were identified in thick-smear blood films stained with giemsa. Stool specimens were examined by Kato Katz concentration technique to identify intestinal helminthic and pathogenic protozoan infections. STIs, malaria, and intestinal parasitic infections were treated at the time of diagnosis according to the Tanzanian Ministry of Health standards for prenatal care.

A blood specimen was collected every six months after randomization for measurement of hemoglobin and T-cell subsets. Thin blood films with Leishman's stain were prepared and examined microscopically. Hypochromasia was classified into 4 levels, coded as absent, 1+, 2+, and 3+. Persons who were diagnosed with severe anemia received management according to standard of care, including treatment for parasitic infections, iron supplementation if indicated, and dietary counseling.

### Statistical Analyses

We examined the relationship of vitamin D status at baseline with mortality, HIV disease progression, and anemia using proportional hazards models [13]. Vitamin D status was defined in two ways: insufficient (<32ng/mL or <80nmol/L) *vs.* adequate and in quintiles based on the distribution of vitamin D levels in this population. The cut-off of 32ng/mL for vitamin D insufficiency was based on requirements for optimal calcium homeostasis [14] and previous studies [15]. We examined the times to: death from AIDS-related causes, all-cause death, progression to WHO stage III or higher, and composite endpoints of mortality and HIV disease progression. For those without the outcomes, follow-up ended on the date on which the stage was last assessed.

The relationship of vitamin D status with T-cell counts was analyzed with generalized estimating equations and SAS Proc Genmod software. Change in T-cell counts between consecutive visits was modeled as a continuous response variable with vitamin D status and other covariates as explanatory variables across the repeated measures in each individual. Robust estimators of the variance were used to construct confidence intervals, using a working correlation matrix with m-dependent structure and m = 1 (assumes correlations between adjacent observations are non-zero and equal). We also examined potential non-linear relationships of change in T-cell counts between consecutive visits with time since randomization non-parametrically with restricted cubic splines [16], [17]. If a non-linear relationship was found, we added the selected cubic spline terms to the above-specified model as covariates with vitamin D status.

We used proportional hazard models to investigate the relationship of vitamin D status with time to the first occurrence of each definition of anemia. Women who had the endpoint of interest at baseline were excluded. Anemia was defined as hemoglobin <110g/L, and severe anemia as hemoglobin <85g/L. We examined the morphology of peripheral red blood cells as a proxy for iron deficiency (hypochromic microcytic cells) and vitamin B-12 and folate deficiency (macrocytic cells) [18]. Hypochromic microcytic anemia was categorized as severe (hypochromasia≥2, microcytosis), moderate and above (hypochromasia≥1, microcytosis), and mild and above (hypochromasia≥1, with or without microcytosis). Macrocytosis was defined as the presence of any macrocytic cells.

We investigated potential non-linear relationship of continuous vitamin D levels with the risk of major outcomes, including HIV disease progression and mortality non-parametrically with stepwise restricted cubic splines [16], [17]. Tests for non-linearity used the likelihood ratio test, comparing the model with only the linear term to the model with the linear and the cubic spline term.

We adjusted for possible confounders of baseline vitamin D levels and age, WHO HIV disease stage and CD4 T-cell count at baseline, and treatment regimen as *a priori* risk factors for the outcomes in all multivariate models, except for change in T-cell counts. This strategy to control confounding uses prior knowledge to identify potential confounders, and includes those that lead to a greater than 10% ‘change-in-estimate’ for the incidence rate ratio of vitamin D status in the model [19]. Baseline T-cell counts were not adjusted for in the multivariate model for change in T-cell counts, since, in analysis of covariance models, it gives biased estimates in non-randomized studies. Observations with missing data for covariates were retained in the analysis using the missing indicator method for variables missing greater than 1% of the observations [20]. Statistical analyses were performed using SAS software version 9.2 (SAS Institute Inc., Cary, NC, US).

## Results

Of the 1078 women enrolled in the trial, baseline vitamin D concentrations were known for 885; one woman was excluded for having WHO HIV stage IV disease. The median follow-up time for participants included in this analysis was 69.5 months (Interquartile Range: 44.7–79.3 months). Baseline characteristics of the 884 women included in this analysis are presented in **Table 1**. T-cell counts measured at least twice during follow-up were available for only 636 women. There was no significant difference in CD4 T-cell counts at baseline between the women with low vitamin D and women with adequate vitamin D (Wilcoxon test; p = 0.29). However, CD8 and CD3 T-cell counts were significantly higher in women with low vitamin D, compared to women with adequate vitamin D (p<0.01, p = 0.02, respectively). The results obtained in analyses with low *vs.* adequate vitamin D status were similar to those obtained with vitamin D quintiles (data not shown), unless noted below.

**Table 1:** Baseline Characteristics of Women enrolled in the trial with available vitamin D levels (N = 884).

	Low Vitamin D *(n = 347)*	Adequate Vitamin D *(n = 537)*
Variable	Mean ± SD* or Frequency (%)	Mean ± SD or Frequency (%)
Age		
Less than 20 years	45 (12.97)	71 (13.22)
20–24 years	144 (41.50)	213 (39.66)
25–29 years	105 (30.26)	166 (30.91)
Greater than or equal to 30 years	53 (15.27)	87 (16.20)
WHO HIV Stage		
I	278 (80.12)	457 (85.10)
II	60 (17.29)	75 (13.97)
III	9 (2.59)	5 (0.93)
CD4 Category, cells/µL		
1 (<200)	46 (13.94)	61 (12.01)
2 (200 to 500)	185 (56.06)	280 (55.12)
3 (> = 500)	99 (30.00)	167 (32.87)
CD3, cells/µL	1282.98±456.36	1209.55±457.99
CD8, cells/µL	808.68±354.06	722.03±317.82
Hemoglobin at baseline, g/L	93.50±17.29	95.25±16.70
Vitamin D [Serum 25(OH)D] at baseline, ng/mL	24.22±6.16	43.07±9.40

* SD: Standard Deviation.

**Figure 1:**
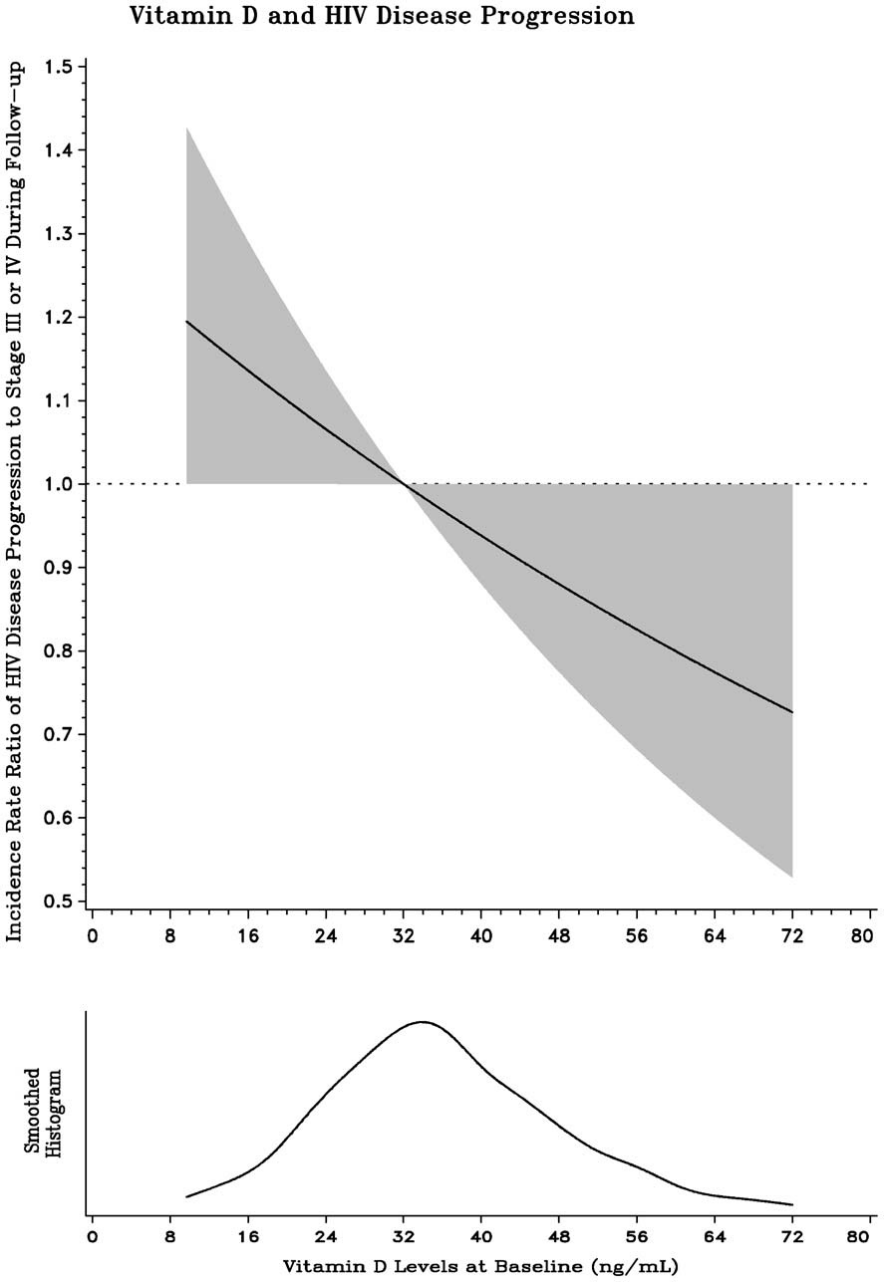
Relationship of Vitamin D status with progression to HIV disease stage III or IV during follow-up.

During follow-up, 287 women died: 201 of AIDS-related causes. 525 women progressed to WHO HIV stage III disease or higher (**Table 2**). After multivariate adjustment, the risk of progression to stage III disease or greater was 1.25 times greater in women with low baseline vitamin D concentrations (95% CI: 1.05, 1.50; p = 0.01), compared to women with adequate vitamin D status. In additional analyses, the relationship between continuous vitamin D levels and risk of disease progression was linear with the risk of disease progression decreasing with increasing vitamin D levels (p = 0.05; **Figure 1**).

**Table 2:** Vitamin D Status and HIV Disease Progression and Mortality among HIV-infected Women.

	Vitamin D					
	Low	Adequate	Univariate Models		Multivariate Models^1^	
Outcome	% (N)^2^	% (N)^2^	RR (95% CI)	P^3^	RR (95% CI)	P^3^
Progression to ≥ HIV disease stage III	60.1 (333)	61.2 (531)	1.20 (1.00, 1.43)	0.05	1.25 (1.05, 1.50)	0.01
Progression to ≥ HIV disease stage III or death from AIDS-related causes	67.8 (338)	66.4 (533)	1.20 (1.02, 1.42)	0.03	1.26 (1.06, 1.49)	0.01
Progression to ≥ HIV disease stage III or death from all causes	71.7 (339)	71.7 (533)	1.19 (1.01, 1.40)	0.04	1.23 (1.04, 1.45)	0.01
Death from AIDS-related causes	22.5 (347)	22.9 (537)	1.04 (0.78, 1.38)	0.79	1.11 (0.83, 1.48)	0.47
Death from all causes	32.9 (347)	32.2 (537)	1.08 (0.85, 1.37)	0.51	1.13 (0.89, 1.43)	0.34

1 All multivariate models adjusted for age, treatment regimen, CD4 T-cell counts at baseline, and HIV disease stage at baseline.

2 % (N): Percentage of cases (Total number).

3 p-values obtained from Cox regression models.

**Figure 2:**
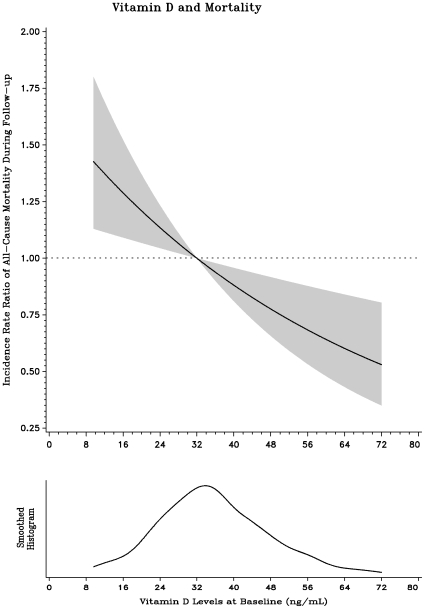
Relationship of vitamin D status with all-cause mortality among HIV-infected women in Tanzania.

There was no relationship observed with either death from AIDS-related causes or death from all causes with the binary variable of low versus adequate vitamin D. However, the women in the highest vitamin D quintile had a 42% lower risk of all-cause mortality compared to women in the lowest quintile (Incidence Rate Ratio [RR]: 0.58; 95% CI: 0.40, 0.84; p<0.01). In analyses with continuous vitamin D levels as the exposure, a significant linear relationship was observed with all-cause mortality; the risk of all-cause mortality declined with increase in vitamin D levels (p<0.01; **Figure 2**).

Although women in the low vitamin D group had significantly higher CD8 and CD3 T-cell counts at baseline, there was no evidence that vitamin D status influenced any further change in CD4, CD8, and CD3 T-cell counts during follow-up (**Table 3**).

**Table 3:** Vitamin D Status and T-cell Subset Counts in HIV-infected Women (n = 636).

	Estimated 6 month change in T-cell counts (95% CI)					
	Time-adjusted models^1^			Multivariate adjusted models^2^		
End point	Low Vitamin D	Adequate Vitamin D	P	Low Vitamin D	Adequate Vitamin D	P
CD4, cells/µL	−70.24 (−99.08, −41.41)	−69.12 (−97.45, −40.80)	0.65	−75.01 (−104.21, −45.81)	−74.43 (−102.82, −46.05)	0.82
CD8, cells/µL	−172.65 (−265.60, −79.69)	−168.18 (−259.90, −76.45)	0.39	−170.48 (−263.50, −77.45)	−166.58 (−258.54, −74.63)	0.46
CD3, cells/µL	−233.07 (−329.03, −137.10)	−227.25 (−322.88, −131.63)	0.39	−240.05 (−335.77, −144.34)	−235.38 (−330.37, −140.40)	0.49

1 Time-adjusted models adjusted for length of interval between two successive measurements and time since randomization.

2 All multivariate models adjusted for age, treatment regimen, and HIV disease stage at baseline.

A significant association was also observed between low vitamin D status and anemia and markers of iron deficiency (**Table 4**). Women with low vitamin D levels at baseline had a 46% higher risk (RR: 1.46; 95% CI: 1.09, 1.96; p = 0.01) of developing severe anemia during follow-up in multivariate models, compared to women with adequate vitamin D levels. The rate ratio for severe hypochromic microcytosis associated with low vitamin D levels at baseline was 2.56 (95% CI: 1.72, 3.79; p<0.01). No significant relationship of vitamin D status was observed with macrocytosis.

**Table 4:** Vitamin D Status and Hematological Outcomes.

	Vitamin D					
	Low	Adequate	Univariate models		Multivariate models^6^	
Outcome	% (N)^2^	% (N)^2^	HR (95% CI)	P^1^	HR (95% CI)	P^1^
Severe Anemia (Hb <85g/L)	38.7 (212)	31.0 (339)	1.44 (1.08, 1.92)	0.01	1.46 (1.09, 1.96)	0.01
Anemia (Hb <110g/L)	92.5 (40)	83.0 (94)	1.18 (0.80, 1.75)	0.40	1.19 (0.77, 1.83)	0.43
Hypochromic microcytosis						
Severe^3^	21.9 (256)	10.5 (391)	2.34 (1.58, 3.45)	<0.01	2.56 (1.72, 3.79)	<0.01
Moderate and above^4^	43.6 (234)	34.8 (399)	1.39 (1.08, 1.80)	0.01	1.45 (1.12, 1.88)	0.01
Mild and above^5^	73.5 (151)	66.3 (261)	1.39 (1.10, 1.77)	0.01	1.41 (1.10, 1.80)	0.01
Macrocytosis	4.5 (269)	4.8 (439)	0.99 (0.49, 2.02)	0.99	1.11 (0.54, 2.28)	0.77

1 p-values are from Cox Regression Models.

2 % (N): Percentage of cases (Total number).

3 Severe: Hypochromasia ≥2+ and microcytic cells observed.

4 Moderate and above: Hypochromasia ≥1+ and microcytic cells observed.

5 Mild and above: Hypochromasia ≥1+.

6 All multivariate models adjusted for age, CD4 T-cell counts, HIV disease stage at baseline, and regime received.

## Discussion

In this study, low vitamin D levels at baseline were significantly associated with increased risk of HIV disease progression, severe anemia, and hypochromic microcytosis in HIV-infected Tanzanian women. Women in the highest quintile of vitamin D also had a significantly lower risk of all-cause mortality compared to women in the lowest quintile of vitamin D. In contrast, no significant association of vitamin D status was observed with AIDS-related mortality or T-cell counts.

There is a paucity of research on the relationship of vitamin D with HIV-related health outcomes, particularly in resource-limited settings. Most of the published studies are cross-sectional comparisons of vitamin D levels in HIV-infected patients compared to HIV-uninfected controls. For example, a small study in Switzerland found no significant difference between 25(OH)D concentrations in 6 patients with advanced AIDS, compared to 10 HIV-uninfected controls [21]. In a study from Germany, 1,25(OH)_2_D and 25(OH)D levels were both significantly lower among men and women with HIV-infection (asymptomatic, on ART), compared to uninfected controls [22]. In a study in Norway, mean serum 1,25(OH)_2_D concentrations were significantly lower in HIV-infected patients, compared to healthy, seronegative blood donors; 1,25(OH)_2_D levels were lowest in symptomatic patients, independent of the presence of opportunistic infections [8].

One small longitudinal study in Norway (n = 53) assessed the association between vitamin D levels and survival among HIV-infected individuals. Patients with 1,25(OH)_2_D levels below 25ng/L at baseline had a shorter survival time than those with normal concentrations, after adjusting for CD4 T-cell counts. The association appeared to be stronger in individuals with CD4 counts less than 50/µL [8].

The protective association of vitamin D against HIV disease progression may be explained by its role in immune function. Vitamin D has been shown to improve phagocytic capacity of macrophages, cell-mediated immunity, and increase natural killer cell number and cytolytic activity, suggesting an important role in response to infections. Laboratory studies have identified several mechanisms by which vitamin D could slow HIV disease progression [7]. For example, 1,25(OH)_2_D decreases CD4 surface receptor expression in promyelocytic leukemia cell line [23] and human monocytes [24]. If confirmed, these results could constitute a mechanism to control viral entry and cytopathogenic effects. In another study, *in vitro* pre-treatment of human peripheral blood monocytes with 1,25(OH)_2_D, but not 25(OH)D, decreased HIV infection by 95% [25]. The recent elucidation of vitamin D's role in the innate immune response *via* toll-like receptors (TLRs) [4], suggests potential modulation of pathogen entry by vitamin D and its metabolites. This mechanism has been shown to be important in the immune system's response to tuberculosis; TLR stimulation of human macrophages up-regulates the expression of VDRs and induces the enzyme (CYP27b1) that catalyzes the conversion of 25(OH)D to 1,25(OH)_2_D, the biologically active metabolite of vitamin D. In presence of adequate 25(OH)D, VDR upregulation leads to cathelicidin induction, an antimicrobial peptide with direct action against intracellular pathogens including *Mycobacterium tuberculosis* [4], a leading cause of disease progression and mortality among HIV-infected patients [26]. Increased resistance to tuberculosis could potentially prolong survival in these patients.

Our study did not find any relationship of vitamin D with change in T-cell counts during follow-up. Though there are several laboratory studies linking vitamin D with T-cell function, no studies have found such an association *in vivo* in HIV-infected patients. It is possible that beneficial effects of vitamin D may be *via* modulation of innate immunity, rather than cell-mediated immunity.

Our finding of a relationship between vitamin D status and risk of severe anemia and iron deficiency could have several potential explanations. Iron deficiency impairs intestinal fat absorption, and may consequently decrease vitamin D absorption [27]. Conversely, vitamin D deficiency could cause anemia *via* increased inflammation or marrow myelofibrosis [28]. An association between low vitamin D status and lower hemoglobin levels or iron deficiency has been observed in earlier studies in individuals with renal disease in NHANES III [29] and in studies in children in minority communities in Britain. For example, in a study in 145 Asian children in Birmingham, the children with low plasma vitamin D concentrations had significantly lower hemoglobin and serum iron concentrations [30]. In Bangladeshi, Pakistani, and Indian communities in England, one-fifth of children surveyed showed signs of both deficiencies; during the winter 50% of children with low vitamin D had low hemoglobin levels (vs. 0% in children with normal vitamin D). Iron deficiency was a significant risk factor for low vitamin D levels in all three ethnic groups [31].

A limitation of the current analysis is the possibility of reverse causation; since vitamin D levels were assessed at baseline when participants were already HIV-infected, the temporal relationship between HIV and vitamin D status could not be ascertained. It is possible that advanced HIV disease may lead to lower serum 25(OH)D levels. However, most participants were stage I disease at baseline. Additionally, controlling for baseline CD4 T-cell counts and HIV disease stage strengthened the observed associations, weakening the possibility of reverse causation.

Another limitation is that the assay for vitamin D assessment does not measure vitamin D_2_, the form of vitamin D obtained through supplements, reliably; however supplementation was unlikely in this population. Additionally, our choice of cut-off for vitamin D is to make results clinically more relevant and comparable with other studies; however, similar effect estimates were obtained for major outcomes such as HIV disease progression and anemia by using population-based quintiles of vitamin D. Further, potential modification of vitamin D status by ART could not be examined, as study participants were not receiving ART.

Our study provides initial support for a potentially beneficial effect of adequate vitamin D status on HIV disease and related outcomes. If confirmed in randomized controlled trials, vitamin D supplementation could be a simple and low cost method to prolong the time to initiation of anti-retroviral therapy in HIV-infected patients, particularly in resource-limited settings. Evidence from larger studies and randomized trials is urgently needed to examine the effects of vitamin D in HIV-infected patients and its interaction with ART.
